# An anonymised longitudinal GPS location dataset to understand changes in activity-travel behaviour between pre- and post-COVID periods

**DOI:** 10.1016/j.dib.2022.108776

**Published:** 2022-11-23

**Authors:** Milton Giovanny Moncayo-Unda, Marc Van Droogenbroeck, Ismaïl Saadi, Mario Cools

**Affiliations:** a Central University of Ecuador, Faculty of Engineering and Applied Sciences, Quito, 170521, Ecuador; b University of Liège, Local Environment Management & Analysis - LEMA (UEE), Liège, 4000, Belgium; c University of Liège, Montefiore Institute of Electrical Engineering and Computer Science, Liège, 4000, Belgium; d Université Gustave Eiffel, IFSTTAR, COSYS-GRETTIA, Marne-la-Vallée, 77420, France; e KULeuven Campus Brussels, Department of Information Management Simulation and Modeling, Brussels, 1000, Belgium; f Hasselt University, Faculty of Business Economics, Hasselt, 3500, Belgium

**Keywords:** Google location history (GLH), Activity point location (APL), GPS, Timeline tracking, Longitudinal data

## Abstract

Collecting GPS data using mobile devices is essential to understanding human mobility. However, getting this type of data is tricky because of some specific features of mobile operating systems, the high-power consumption of mobile devices, and users’ privacy concerns. Therefore, data of this kind are rarely publicly available for scientific purposes, while private companies that own the data are often reluctant to share it. Here we present a large anonymous longitudinal dataset of Activity Point Location (APL) generated from mobile devices’ GPS tracking. The GPS data were collected by using the Google Location History (GLH), accessible in the Google Maps application. Our dataset, named AnLoCOV hereafter, includes anonymised data from 338 persons with corresponding socio-demographics over approximately ten years (2012–2022), thus covering pre- and post-COVID periods, and calculates over 2 million weekly-classified APL extracted from approximately 16 million GPS tracking points in Ecuador. Furthermore, we made our models publicly available to enable advanced analysis of human mobility and activity spaces based on the collected datasets.

**Specifications Table**

**Table:** 

Subject	Social Sciences, Geography
Specific subject area	Mobility, Transportation, Activity-travel.
Type of data	GPS data, Table
How the data were acquired	The data is based on Google Timeline. Each person used the Google Maps application on their mobile device to get the Google Location History (GLH) data. Each person requested the file from Google and shared it with us. After receiving the file, a recruitment questionnaire to collect some additional socio-demographic information was performed. All data was stored under strict ethical and privacy terms. The questionnaire is available as supplementary material.
Data format	Raw, Filtered, Anonymised
Description of data collection	We organised an information session to inform details of the project and to explain participants on how we would use it and the treatment we would give to the data. Participants were recruited on a voluntary basis and only adults (age 18+) participated in the study. To ensure the privacy of the participants, all data was anonymised, and the original data was destroyed after transformation and processing.
Data source location	• **Institution:** Central University of Ecuador • **City/Town/Region:** Quito/Quito/Pichincha • **Country:** Ecuador
Data accessibility	**Repository name:** Mendeley Data **Data identification number:** https://doi.org/10.17632/vk77k9gvg3.2 **Direct URL to data:** https://data.mendeley.com/datasets/vk77k9gvg3 **Direct URL to code:** https://gmoncayocodes.github.io/ActivityPointLocationGenerator/ **Direct URL to survey:** https://github.com/GmoncayoCodes/ActivityPointLocationGenerator/blob/c0148c7ca469648db722b6a2140a6d112b6b8856/demographic/Survey_EN.pdf

## Value of the Data

• AnLoCOV contributes to the scientific community in the domain of urban/human mobility behaviour by making an anonymised longitudinal GPS dataset (N=338) from Google Maps openly available. AnLoCOV includes Activity Point Locations over long PRE- and POST-COVID periods, allowing a deeper insight into the longitudinal effects of the COVID-19 pandemic on the activity space/time dimensions. Additionally, GPS tracking, summary statistics and socio-demographic information on participants are provided if more advanced travel behaviour analyses are desired.
• AnLoCOV provides relevant and validated data to support urban/human mobility research. The spatial analysis of Activity Point Locations and Human Activity Spaces clarifies the relationships between the built environment/the transport system and travel behaviour in cities.
• AnLoCOV is publicly available at Mendeley Data [1]. We provide algorithms entirely based on open-source frameworks and make them publicly available on GitHub [2]. The methodological workflow can be re-used with JSON data from other applications or geographical locations.

## 1 Data Description

Nowadays, mobile devices’ ubiquity and affordability of smartphone technology increase the possibility of getting, in a secure, efficient, and inexpensive way, human movement data using GPS [3]. The use of this type of data has been growing in many studies related to mobility patterns [4,5], route choice modelling [6,7], transport mode recognition [8,9], origin-destination trip purpose [10,11], identification of activity stops locations [12,13,14], sports activity identification [15], and in the human activity behaviour analysis [16,17]. This new data collection framework allows the collection of considerable amounts of data compared with traditional methods [18], which grants in-depth and long-term research of Human Activity Spaces (HAS) [19].

Places frequently visited by people represent the Activity Point Locations (APL). People spend time in these places doing daily activities (e.g., home, work, supermarket, bus stop, gas station, traffic jam, park, church, cinema, and others). These points are also well-known as Points of Interest (POI) and are the basis for measuring the size of HAS [20]. The APL identification based on mobile devices’ GPS tracking has improved because of innovative spatial analysis software packages. These analyses can include day-to-day activity-travel variability for estimating activity-based models of travel demand and the complexity of persons’ daily activity-travel patterns (number of stops, activity-travel sequences). However, the main problem in the scientific community is to share this data publicly due to people's privacy [21], so it is essential to anonymise it to share data for further research. The anonymisation technique must enable data access while maintaining people's privacy and keeping the data structure to analyse it efficiently within the original research purpose [22,23] despite the undeniable semantic information loss [24]. Empirical APL data collected on a longitudinal basis are rarely publicly available, mainly because of the costs and difficulty of acquiring data over a long period of time [25].

AnLoCOV is an open, anonymised, and longitudinal dataset with spatial APLs computed on a weekly basis. This dataset stores information collected using GLH, which, once activated, accumulates GPS data from the mobile device and can be used as a mobility data acquisition tool [26].

**Fig. 1:**
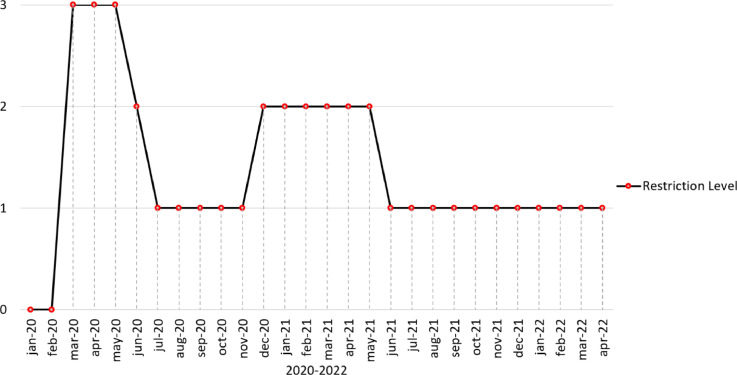
Different restriction levels imposed by the government of Ecuador due to COVID-19. Their meaning is as follows: 0 = no restrictions, 1 = light restrictions, 2 = moderate restrictions, 3 = full restrictions

In addition, AnLoCOV considers different restriction levels imposed by the government of Ecuador due to COVID-19 from March 2020, when practically all cities in the world were in lockdowns to reduce mobility and prevent the spread of the disease. The Ecuadorian emergency operations committee (EOC) periodically analysed the country's health conditions. The lowest level (0) implies no restrictions, i.e., before COVID-19. The highest level (3) implies total lockdown; only priority public services such as health, food and transport are provided. The intermediate levels (1 and 2) imply vehicular prohibition during certain days of the week, curfew during the nights and capacity control in enclosed or crowded places. These restriction levels are depicted in Fig. 1. and encoded in the datasets (see Data Description section).

AnLoCOV encompasses four anonymised datasets distributed in CSV format:

• **DemographicData:** Contains anonymous demographic data from 338 persons. The data is ordered by Google Location History id.
• **GPSTrackingData:** Contains more than 16 million GPS point coordinates corresponding to the clean GPS tracking of each person. The anonymisation of this dataset is based on the gravity point of the whole GPS data. The data is ordered by Google Location History id and datetime.
• **APLData:** Contains more than 2 million Activity Point Location coordinates, including cluster information. The anonymisation of this dataset is based on the most visited Activity Point Location (cluster labelled as 0). The data is ordered by Google Location History id, week and trip.
• **SummaryData:** Contains summary measures of GPSTrackingData and APLData, like the number of GPS, APL, and clusters. It is ordered by Google Location History id, week, and trip.

All datasets are publicly available and licensed with the Creative Commons BY 4.0 license, which allows their use for any purpose (including commercial use) if appropriate credit for the dataset is declared.

A detailed description of data records is given in Tables 1, 2, 3 and 4.

**Table 1:** Description of the DemographicData records

Dataset	Column	Description
**Demographic Data**	id	GLH File identifier, one per person (Text)
	universityMember	Person is a university member of the Central University of Ecuador (Text)
	age	Person age (Number)
	gender	Person is Male or Female (Text)
	homeLocation	Person home location is North, Centre, South, Valley, or Out of Quito (Text)
	vehicleOwner	Person has or not a private vehicle (Text)
	usualTransportPattern	Person usual transport pattern is Car or Public Transportation & Others (Text)
	futpPublicTransport	Usual transportation pattern's frequency in the last 12 months at the time of the socio-demographic survey (Number) 0 – Never, 1 – Once a year, 2 – Once a month, 3 – Once a week, 4 – One to five times per week, 5 – All days
	futpCarDriver	
	futpCarPassenger	
	futpTaxi	
	futpMotorcycle	
	futpBicycle	
	futpOnFoot	
	futpPlane	
	fuaStudies	Usual activity frequency in the last 12 months at the time of the socio-demographic survey (Number) 0 – Never, 1 – Once a year, 2 – Once a month, 3 – Once a week, 4 – One to five times per week, 5 – All days
	fuaWork	
	fuaSports	
	fuaEntertainment	
	fuaShopping	
	fuaHome

**Table 2:** Description of the GPSTrackingData records

Dataset	Column	Description
**GPS** **Tracking** **Data**	id	GLH File identifier, one per person (Text)
	datetime	UTC date and time of GPS track point (Text)
	lat	GPS point latitude in WGS84 decimal degrees (Number)
	lon	GPS point longitude in WGS84 decimal degrees (Number)
	covidStatus	GPS point Binary COVID status identifier (Number)
	restrictionLevel	GPS point Restriction level identifier (Number)

**Table 3:** Description of the APLData records

Dataset	Column	Description
**APL** **Data**	id	GLH File identifier, one per person (Text)
	idWeek	Week data identifier (Text)
	idTrip	Trip per week data identifier (Number)
	datetime	APL UTC date and time (Text)
	lat	APL latitude in WGS84 decimal degrees (Number)
	lon	APL longitude in WGS84 decimal degrees (Number)
	cluster	APL cluster identifier (Number)
	covidStatus	APL Binary COVID status identifier (Number)
	restrictionLevel	APL Restriction level identifier (Number)

**Table 4:** Description of the SummaryData records

Dataset	Column	Description
**Summary** **Data**	id	GLH File identifier, one per person (Text)
	idWeek	Week data identifier (Text)
	idTrip	Trip per week data identifier (Number)
	GPSPoints	Trip total GPS points (Number)
	APLs	Trip total APLs (Number)
	clusters	Trip total clusters (Number)
	covidStatus	Trip Binary COVID status identifier (Number)
	restrictionLevel	Trip Restriction level identifier (Number)

## 2 Experimental Design, Materials, and Methods

**Fig. 2:**
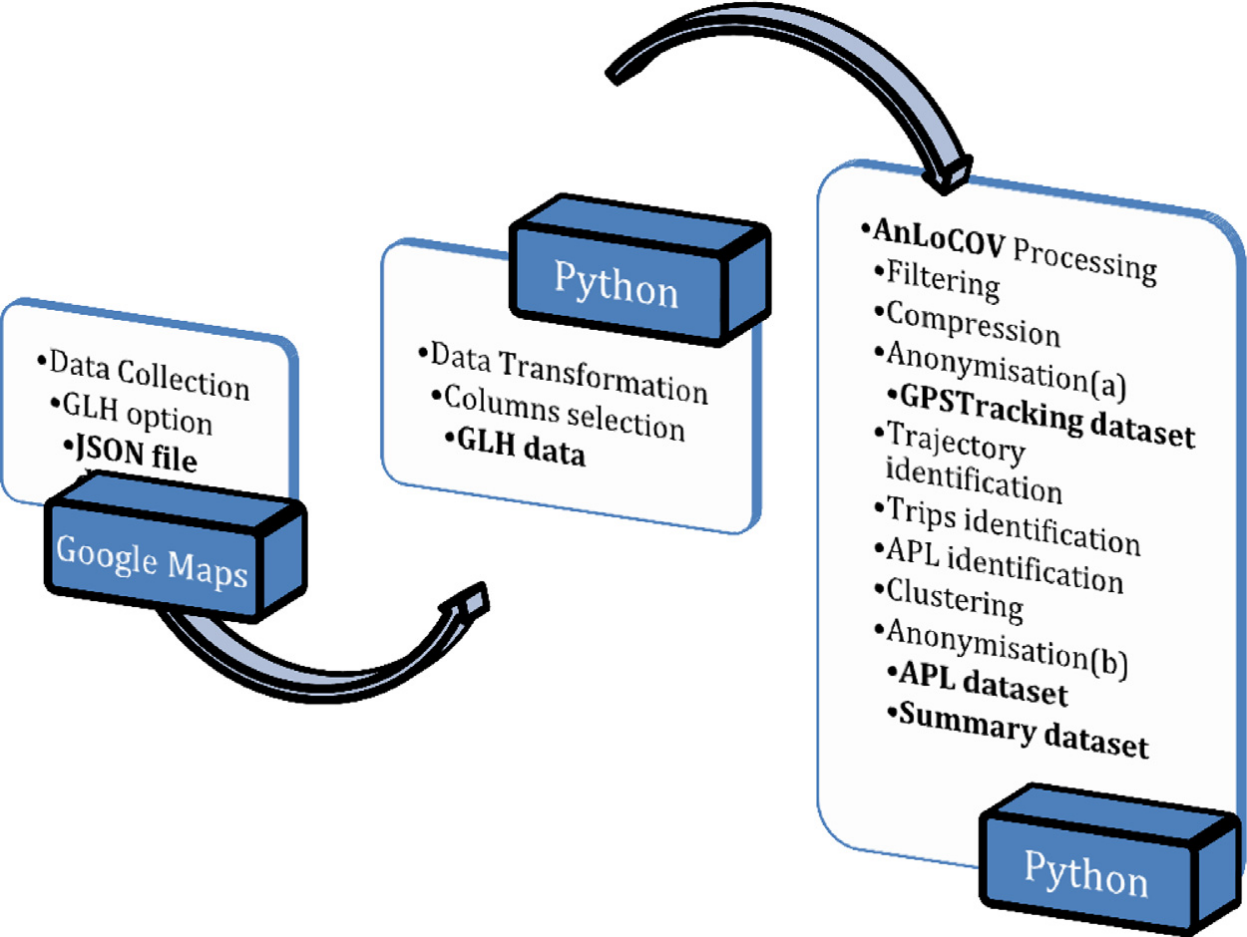
Schematic overview of the data preparation process. The data is first collected in JSON files. Then, it is transformed into a proper format before being processed and anonymised

A schematic overview of the process adopted for data preparation is given in Fig. 2.

The data stem from adult participants who provided informed consent and agreed to share their data anonymously. The data preparation work presented below ensures compliance with GDPR and University's ethical committee regulations. The data preparation framework includes four successive stages: data collection, data transformation, AnLoCOV processing and demographics survey.

### 2.1 First stage: data collection

Data is an essential component in the research process. We use the GLH component of the Google Maps platform, an innovative and widely used application, to get location data worldwide. Through GLH, Google Maps collects the device's locations via GPS, Wi-Fi, or mobile network connections if the GPS is active. The location data coordinates are defined in accordance with the WGS84 coordinate reference system.

The device's hardware, operating system version, or use in indoor locations (e.g. tunnels, buildings) can result in location data loss accuracy. However, Macarulla Rodriguez, in his paper [27], demonstrates that this loss of accuracy is not significant, so we can assume that the device was close to a specific location. Also, a continuous Internet connection, GPS enabled, and frequent use of the Google Maps application to search for routes or move from one place to another improve the location data accuracy. The drawback is that continued use of the device's GPS may result in battery performance problems.

Once the GLH is activated in the Google account, Google tracks, when possible, the GPS-based mobility data from the mobile device. Each person can manage their location history status (pause, disable, edit, or delete) in Google Timeline. By default, GLH is disabled in all Google accounts.

**Fig. 3:**
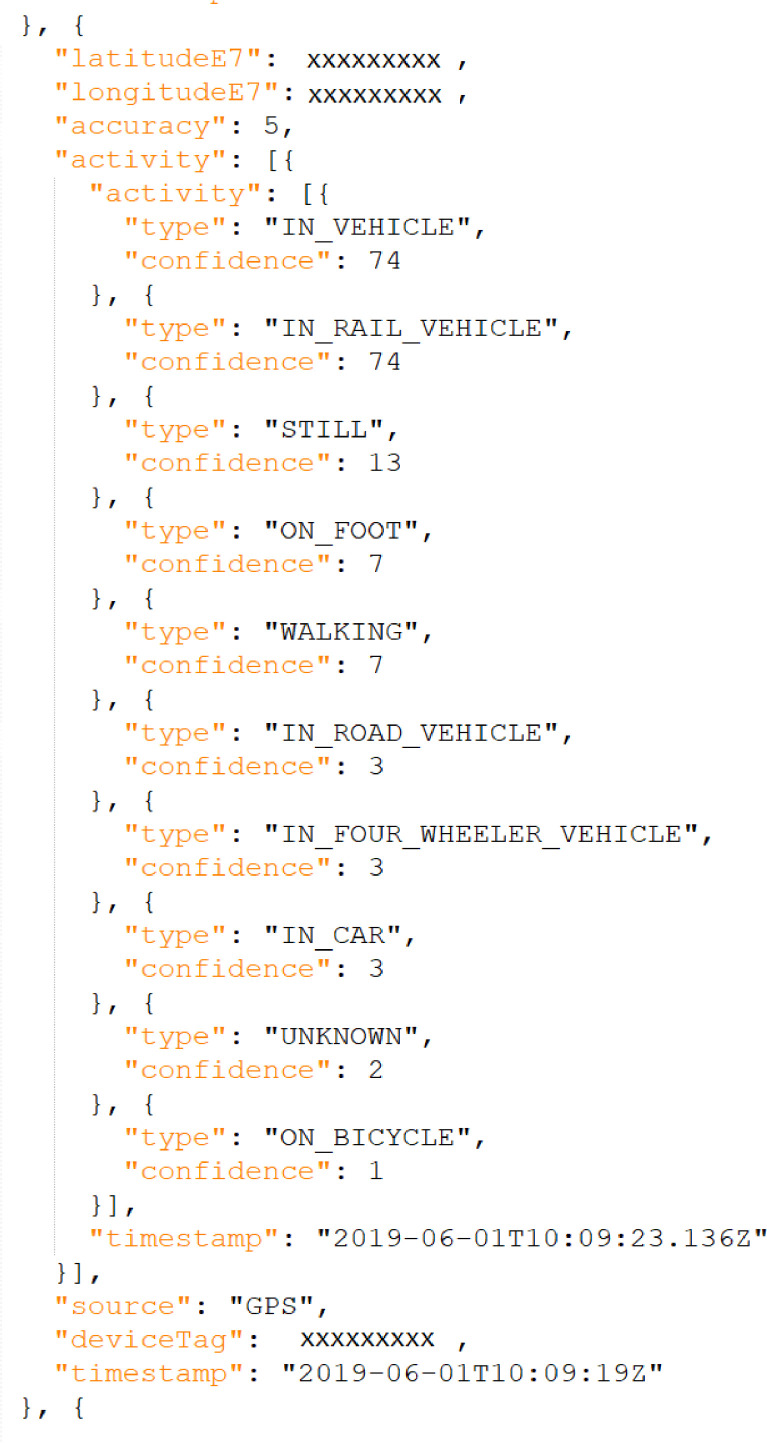
Extract of a GLH JSON file used to build our dataset. Some data has been replaced by “xxxxx” on purpose

The last step in this stage is to request the GLH JSON data from Google via the Google Takeout application. This activity was carried out by each participant of the study. Fig. 3 shows an extract of a JSON data file provided by Google. This file is the input for the next stage.

### 2.2 Second stage: data transformation

A JSON file transformation is required to manipulate data computationally. In this paper, we assume the most straightforward possible representation of location data: each observation consists of a timestamp and a location point. We will use three fields of the GLH JSON file to represent data: (1) timestamp (recorded UTC date and time), (2) latitudeE7, and (3) longitudeE7 (recorded WGS84 GPS location coordinates). An algorithm in Python [28] will transform the original GLH JSON file into a comma-separated value (CSV) file with three columns (datetime, latitude and longitude). This file is then the input for the AnLoCOV processing stage.

### 2.3 Third stage: AnLoCOV processing

**Fig. 4:**
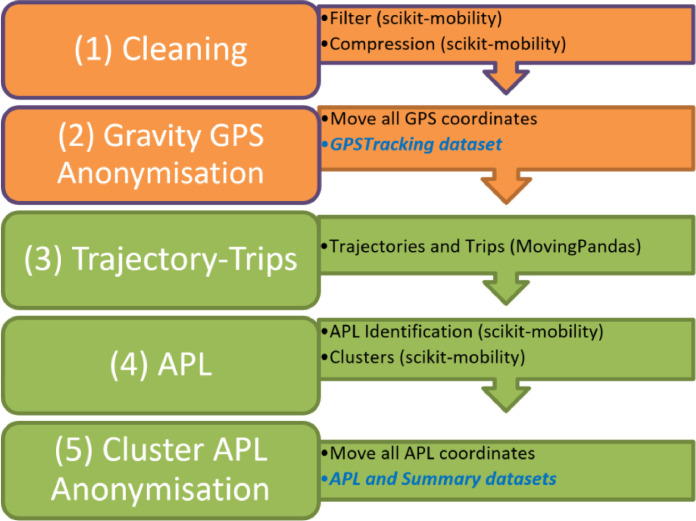
Workflow for generating processed and anonymised activity point locations. The names of the used Python packages are given between parentheses. In blue Italic, the generated datasets

Fig. 4 represents the generic workflow for AnLoCOV processing in Python. Specific pre-processing steps were applied to (1) clean the GLH data, (2) anonymise GLH data based on a gravity point, (3) identify the trajectories and trips, (4) compute APLs and clusters, and finally (5) anonymise APL data based on a cluster point for sharing.

#### 2.3.1 (1) Data cleaning

The presence of noise in the data is a consequence of the accuracy loss mentioned earlier in the data collection stage. Therefore, we apply a filtering process that deletes GPS points considered noise or outliers in the trajectory. For example, when two consecutive GPS points have much higher speeds than the globally defined speed limits in urban or non-urban areas, the second GPS point is considered an outlier and is subsequently removed from the dataset.

A compression step further reduces the number of GPS points while preserving the trajectory properties. It works as follows. When the Euclidean radius distance is very small between consecutive points, it implies the points are in a very close neighbourhood of the same location. Subsequently, all these points are merged into a single point whose location is given by the median of all point coordinates, while the associated timestamp corresponds to the first point. This process results in significant compression of the number of GPS locations.

The set of parameters for this step is defined in Table 5.

**Table 5:** Data cleaning parameters.

Data Cleaning	Parameter (value)	Description
Filter	max_speed_kmh (200)	Delete a GPS point if the speed from the previous point is higher than 200km/h
Compression	spatial_radius_km (0.05)	Compress consecutive GPS points if the Euclidean radius distance between points is less than 0.05km (50m)

#### 2.3.2 (2) Gravity GPS anonymisation

After data filtering and compression, we obtain the clean GPS points. At the end of this second step, we release the first dataset **(GPSTrackingData)**. Nevertheless, to guarantee privacy, we need to anonymise the GPS locations. This is done as follows: Data is anonymised based on the gravity point calculated for each person's latitude and longitude data. The gravity point is set at the location 0-latitude and 0-longitude, and all GPS points are translated accordingly. This translation preserves the original shape position and distances between GPS points.

While this first dataset is the core of AnLoCOV, we provide further datasets to ease the analysis at higher levels, which is at the level of trips and Activity Point Locations (APLs). It is important to note that we release all the codes for the different steps, allowing any researcher to adapt the parameters to their needs for their own data, while providing an example of how to deal with higher-level GPS locations. However, trips and APLs must be calculated on the original, non-anonymised GPS locations because, at higher levels, we are interested in cross-persons analyses. Therefore, we need to anonymise the APLs as well before releasing the subsequent datasets.

#### 2.3.3 (3) Trajectory-trip identification

All consecutive GPS points with a minimum length are converted into weekly trajectories for trajectory-trip identification. The process excludes short trajectories. These weekly trajectories are split into trips with a minimum gap threshold and a minimum length.

The set of parameters for this step is defined in Table 6.

**Table 6:** Trajectory-trip identification parameters.

Trajectory-Trip identification	Parameter (value)	Description
Trajectory	min_length (200)	The minimum length for trajectories is 200m
Trip	gap (30)	The minimum gap time in a trajectory to split into trips is 30min
	min_length (100)	The minimum length for trips is 100m

#### 2.3.4 (4) APL identification and clustering

This step detects APLs for each trip. When the person stays a minimum number of minutes within a Euclidean radius distance from a given GPS point location during the trip, it forms an APL. The APL's time is the time of the initial GPS point, and the coordinates are the median latitude and longitude values of all the GPS points found within the specified distance.

The clustering step ranks all APLs. Each APL is labelled with a clustering number depending on its spatial proximity and the number of visits to similar locations at different times. Density-based spatial clustering analysis is conducted by using the DBSCAN algorithm. Each cluster is sequentially labelled, starting from 0, whereas cluster 0 corresponds to the most visited APL over time.

The set of parameters for this step is defined in Table 7.

**Table 7:** APL identification and clustering parameters.

APL identification and Clustering	Parameter (value)	Description
APL Identification	minutes_for_a_stop (5)	The minimum time spend in a place to consider it as a stop is 5min
	stop_radius_factor (1)	The minimum Euclidean radius distance for a stop is 1Km (1000m) stop_radius_factor*spatial_radius_km
	spatial_radius_km (1)	
Cluster	cluster_radius_km (0.05)	The minimum radius proximity of the points in each cluster is 0.05km (50m)
	min_samples (1)	The minimum GPS points for a Cluster is 1

#### 2.3.5 (5) Cluster APL anonymisation

The latitude and longitude coordinates of each APL are anonymised. In this case, the reference point for anonymising the coordinates of each person is set as the most visited APL over time (cluster labelled as 0). In other words, we compute the mean latitude and longitude of the APLs clustered as 0 and translate all APLs accordingly. The most visited APL ends at the location 0-latitude and 0-longitude. This translation preserves the original shape position and distances between each APL.

Finally, we summarise trip measures such as the total number of GPS points, APLs, and clusters per trip. We do not consider the geodesic distances because it would provide some information about the exact geographical map and contradict our willingness to anonymise the data fully. After this step, we obtain the second and third datasets, i.e. **(APLData)** and **(SummaryData)**, respectively.

### 2.4 Fourth stage: demographics survey

After validating and processing GLH data, each volunteer participant was asked to give socio-demographic information by answering an online survey via Google Forms [29] and providing informed consent to use and share their anonymised GLH data. Only consenting participants are included in AnLoCOV. A copy of the survey is attached as supplementary material. After this stage, we obtain the last dataset (**DemographicData)**

## Ethics Statements

All data has been anonymised to respect the privacy of participants. Informed consent was obtained from each one by completing the demographic data survey. In addition, because we used data from Google, the Board for Ethics and Scientific Integrity of the University of Liège confirmed that the project meets the standard ethical requirements and complies with the GDPR. The protocol number is JUR26262.

## CRediT authorship contribution statement

**Milton Giovanny Moncayo-Unda:** Investigation, Writing – original draft, Conceptualization, Methodology, Software, Data curation, Formal analysis, Validation. **Marc Van Droogenbroeck:** Writing – review & editing, Conceptualization, Methodology, Supervision. **Ismaïl Saadi:** Writing – review & editing, Conceptualization, Methodology, Supervision. **Mario Cools:** .

## Declaration of Competing Interest

The authors declare that they have no known competing financial interests or personal relationships that could have appeared to influence the work reported in this paper.

## Data Availability

AnLoCOV (Original data) (Mendeley Data).

## Appendix Supplementary Materials

Image, application 1

Image, application 2

Image, application 3
