# Supplementary material for: An anonymised longitudinal GPS location dataset to understand changes in activity-travel behaviour between pre- and post-COVID periods
Source: Data Brief. 2022 Nov 23;45:108776. doi: 10.1016/j.dib.2022.108776 (PMC9747621; doi:10.1016/j.dib.2022.108776)
Supplement: Supplementary file 1 [file mmc1.pdf]

Université de Liège

Information relating to the processing of personal data

Multidimensional GPS data framework on mobility

The project manager(s) will take all necessary measures to protect the confidentiality and security of your personal data (or those of the person for whom you have legal responsibility), in accordance with the General Data Protection Regulation (GDPR - EU 2016/679) and the Law of 30 July 2018 on the protection of individuals with regard to the processing of personal data.

1. Who is the controller?

The Data Controller is the promoter of the study, namely the University of Liège, whose headquarters is located at Place du 20-Août, 7, B- 4000 Liège, Belgium.

The internal investigator is the investigator whose name appears on the informed consent form.

2. What data will be collected?

The investigator will collect identification data (email address, gender, date of birth, income, GPS locations). He undertakes to collect only the data strictly necessary and relevant to the purposes pursued.

3. For what purpose(s) will this data be collected?

The data is collected for the following purposes:

- PhD theses;
- Publication of articles presenting the results of the study in specialized journals;
- Communication of the results of the study during conferences;
- Re-use of data collected for the purpose of carrying out complementary studies in the same field of research.

The results of this study will be systematically anonymized before any dissemination.

4. How long will this data be kept and by whom?

The identification data will be kept by the investigator until the anonymization phase. At the end of this phase, the data will be destroyed (deletion of the database containing this data). This will make the research data completely anonymous.

5. *How will data be collected and protected during the study?*

The data will be collected through the following phases:

Collection: Collection of identification data by one of the investigators associated with the study. A study-specific code number is given for each participant, which will be used in subsequent phases.

Storage: Identification data are stored in two different databases: Computer and external hard disk. Each one is password protected.

Anonymization: Identification data are anonymized, and the original data are deleted.

Processing: Statistical processing of anonymous results.

Results: Publication and dissemination of anonymous results.

6. *Will these data be made anonymous or pseudo-anonymous?*

The investigator has a duty of confidentiality regarding the data collected. This means that we will never reveal names in the context of a publications or conferences. The investigator will also code the data (replacing identity by an identification code) before any further processing.

The investigator and his team will therefore be the only ones able to establish a link between the anonymous and the identification data. The personal data transmitted will not contain any association of elements that could identify participants.

7. *Who will be able to access and use this data?*

The investigator

8. *Will this data be transferred outside the University?*

No, this data will not be transferred or processed by third parties.

9. *On what legal basis will these data be collected and processed?*

The collection and use of personal data (or the person for whom you have legal responsibility) is based on the University's mission of public interest (RGPD, Art. 6.1.e) and, on the need to process such data for scientific research purposes (RGPD, Art. 9.2.j).

10. *What rights does the person whose data is used have?*

As provided for in the GDPR (Art. 15 to 23), each data subject may, by proving his identity, exercise a series of rights:

- to obtain, free of charge, a copy of the personal data concerning her/him processed in the context of this study and, where appropriate, any available information on their purpose, origin and destination;
- to obtain, free of charge, the rectification of any inaccurate personal data concerning her/him and to obtain that incomplete data be completed;
- to obtain, subject to the conditions provided for by the regulations and free of charge, the limitation of the processing of personal data concerning her/him;
- to oppose, subject to the conditions provided for by the regulations and without charge, for reasons relating to his or her specific situation, the processing of personal data concerning him or her;

- to lodge a complaint with the Data Protection Authority (https://www.dataprotectionauthority.be/, contact@apd-gba.be).

11. How to exercise these rights?

To exercise these rights, you may contact the person(s) responsible for the research project (mgmoncayo@uliege.be, mmoncayo@uce.edu.ec) or the University Data Protection Officer, either by email (pfpirlet@uliege.be) or by letter dated and signed at the following address:

University of Liège
Mr Pierre-François Pirlet,
Bât. B9 Cellule "GDPR",
Quartier Village 3,
Boulevard de Colonster 2,
4000 Liège, Belgique.

Firmado electrónicamente por:
MILTON GIOVANNY MONCAYO UNDA
