# Supplementary material for: An anonymised longitudinal GPS location dataset to understand changes in activity-travel behaviour between pre- and post-COVID periods
Source: Data Brief. 2022 Nov 23;45:108776. doi: 10.1016/j.dib.2022.108776 (PMC9747621; doi:10.1016/j.dib.2022.108776)
Supplement: Supplementary file 2 [file mmc2.pdf]

GPS data collection survey

Dear participant,

I am pleased to extend you a sincere thank you for your collaboration in data collection through the Google Timeline using your mobile device.

I remind you that the data provided will be used exclusively in a research project related to Mobility and Transport in the city of Quito, this project is hosted between the Central University of Ecuador and the University of Liège - Belgium, where I am doing my PhD.

I invite you to answer this short survey which will take approximately 5 minutes and which will provide me with additional information for the human activity spaces study.

In the "Email" field, please enter the email address where you received the link to this survey, only in this way I can validate the successful completion of the process.

Thank you very much for your time and assistance.

Sincerely yours,
Giovanny Moncayo
PhD Student

---

*Required

1. Email *

____

2. Do you agree that you have been informed about the project and that your data will be used for academic research purposes only? *

Mark only one oval.

- ( ) Yes   *Skip to question 3*
- ( ) No   *Skip to section 3 (Informed Consent)*

Demographic Survey

3. 1. Are you student, or staff of the Universidad Central del Ecuador? *

Mark only one oval.

- ( ) Yes
- ( ) No

4. 2. Date of birth: *

____

Example: 7 January 2019

5. 3. Age (years): *

____

6. 4. Gender: *

Mark only one oval.

- ( ) Female
- ( ) Male

7. 5. Home location: *

Mark only one oval.

- ( ) North
- ( ) Centre
- ( ) South
- ( ) Valley
- ( ) Out of Distrito Metropolitano de Quito

8. 6. Do you have an own transportation mode? *

Mark only one oval.

- Yes
- No

9. 7. What transportation mode do you usually use for long distances? (To go from home to work, to the university or to the shopping centre). *

Mark only one oval.

- Public Transportation
- Own Vehicle
- Motorcycle
- Bicycle
- On foot
- Other: ______________________

10. 8. How often have you used the following transport modes in the last 12 months?

Mark only one oval per row.

	All days	One to five times per week	Once a week	Once a month	Once a year	Never
**Public transport**	◯	◯	◯	◯	◯	◯
**Private car as driver**	◯	◯	◯	◯	◯	◯
**Private car as a passenger**	◯	◯	◯	◯	◯	◯
**Taxi**	◯	◯	◯	◯	◯	◯
**Motorcycle**	◯	◯	◯	◯	◯	◯
**Bicycle, Scooter**	◯	◯	◯	◯	◯	◯
**On foot (more than 10 minutes)**	◯	◯	◯	◯	◯	◯
**Plane**	◯	◯	◯	◯	◯	◯

11. 9. How often have you done the following activities in the last 12 months?

Mark only one oval per row.

	All days	One to five times per week	Once a week	Once a month	Once a year	Never
**Studies**	◯	◯	◯	◯	◯	◯
**Work**	◯	◯	◯	◯	◯	◯
**Sports**	◯	◯	◯	◯	◯	◯
**Entertainment**	◯	◯	◯	◯	◯	◯
**Shopping**	◯	◯	◯	◯	◯	◯
**Home activities**	◯	◯	◯	◯	◯	◯

Informed Consent

If you do not agree that you have been informed about the use of your data, you will not be able to complete the process.

I take this opportunity to provide you information about the project:

My name is Giovanny Moncayo, I am a lecturer in the Faculty of Engineering and Applied Sciences at the Central University of Ecuador.

Since 2018 I am doing my PhD at the University of Liège in Belgium, where I have been working on a multidisciplinary research project related to Mobility and Transportation using mobile devices.

The data collected through your Google timeline, will allow us to search for patterns to identify different human activity spaces (For example: home, work, a study centre, a supermarket, a shopping centre, a football stadium, a bus stop, etc, etc, etc...). Our objective is to measure these human activity spaces and understand how they have changed before and after the COVID-19 in order to contribute to improving mobility, urban planning and transport in Quito.

If after having read the above paragraphs you still consider that you have not been informed, your participation in the project will not be considered. If, on the other hand, you agree that you have understood how your information will be used, I invite you to return to the previous question and select the YES option to access the survey.

Thank you very much for your cooperation

Giovanny Moncayo

This content is neither created nor endorsed by Google.

Google Forms
