# Supplementary material for: An anonymised longitudinal GPS location dataset to understand changes in activity-travel behaviour between pre- and post-COVID periods
Source: Data Brief. 2022 Nov 23;45:108776. doi: 10.1016/j.dib.2022.108776 (PMC9747621; doi:10.1016/j.dib.2022.108776)
Supplement: Supplementary file 3 [file mmc3.pdf]

Encuesta recolección de datos GPS

Estimado participante,

Permítame extenderle un sincero agradecimiento por su colaboración en la recolección de datos usando su dispositivo móvil a través de la opción Tus Rutas (Timeline) de Google.

Le recuerdo que los datos proporcionados serán utilizados exclusivamente en un proyecto de investigación relacionado con la Movilidad y el Transporte en la ciudad de Quito, entre la Universidad Central del Ecuador y la Universidad de Lieja - Bélgica, donde me encuentro realizando estudios de Doctorado.

Para finalizar el proceso, le invito a responder esta breve encuesta que le llevará aproximadamente 5 minutos completarla y la cual me proporcionará información adicional para el estudio.

En el campo "Correo electrónico", por favor ingrese la dirección de email donde recibió el enlace de esta encuesta, solo de esa forma podré validar la culminación satisfactoria del proceso.

Agradezco mucho su tiempo y la ayuda prestada.

Atentamente,
Ing. Giovanny Moncayo
Estudiante de Doctorado

---

*Obligatorio

1. Correo electrónico *

____

2. Acepta haber sido informado acerca del proyecto y que sus datos serán utilizados únicamente con fines académicos de investigación? *

Marca solo un óvalo.

- ◯ SI *Ir a la pregunta 3*
- ◯ No *Ir a la sección 3 (Consentimiento Informado)*

Encuesta Datos Demográficos

3. 1. ¿Es Usted Estudiante, Administrativo o Docente de la Universidad Central del Ecuador? *

Marca solo un óvalo.

- Si
- No

4. 2. Fecha de nacimiento: *

____

Ejemplo: 7 de enero de 2019

5. 3. Edad (años cumplidos): *

____

6. 4. Género: *

Marca solo un óvalo.

- Mujer
- Hombre

7. 5. Sector donde se encuentra su residencia: *

Marca solo un óvalo.

- Norte
- Centro
- Sur
- Valles
- Fuera del Distrito Metropolitano de Quito

8. 6. ¿Dispone de un medio de transporte propio para movilizarse? *

Marca solo un óvalo.

- Si
- No

9. 7. ¿Cuál es el medio de transporte que usa habitualmente para recorrer largas distancias? (Ej: Para ir de la casa al trabajo, a la universidad o al centro comercial) *

Marca solo un óvalo.

- Transporte público
- Vehículo particular
- Motocicleta
- Bicicleta
- A pie
- Otros: ____________________

10. 8. ¿Con qué frecuencia ha utilizado los siguientes medios de transporte en los últimos 12 meses?

Marca solo un óvalo por fila.

	Todos los días	Entre 1 a 5 veces por semana	Al menos 1 vez por semana	Al menos 1 vez al mes	Al menos 1 vez al año	Nunca
**Transporte público**						
**Vehículo particular como conductor**						
**Vehículo particular como pasajero**						
**Taxi**						
**Motocicleta**						
**Bicicleta, Scooter**						
**A pie (más de 10 minutos)**						
**Avión**						

11. 9. ¿Con qué frecuencia ha realizado las siguientes actividades en los últimos 12 meses?

Marca solo un óvalo por fila.

	Todos los días	Entre 1 a 5 veces por semana	Al menos 1 vez por semana	Al menos 1 vez al mes	Al menos 1 vez al año	Nunca
**Estudios**	⬭	⬭	⬭	⬭	⬭	⬭
**Trabajo**	⬭	⬭	⬭	⬭	⬭	⬭
**Deportes**	⬭	⬭	⬭	⬭	⬭	⬭
**Entretenimiento**	⬭	⬭	⬭	⬭	⬭	⬭
**Compras**	⬭	⬭	⬭	⬭	⬭	⬭
**Actividades del hogar**	⬭	⬭	⬭	⬭	⬭	⬭

Consentimiento Informado

Si no acepta haber sido informado acerca del uso de sus datos, no podrá completar el proceso.

Aprovecho esta oportunidad para proporcionarle rápidamente información acerca del proyecto:

Mi nombre es Giovanny Moncayo, soy docente en la Facultad de Ingeniería y Ciencias Aplicadas en la Universidad Central del Ecuador.

Desde el año 2018 me encuentro realizando estudios de doctorado en la Universidad de Lieja en Bélgica, donde he venido trabajando en un proyecto de investigación multidisciplinario relacionado con la Movilidad y el Transporte utilizando dispositivos móviles.

Los datos recolectados mediante su timeline de Google, nos permitirán realizar la búsqueda de patrones para identificar diferentes espacios de actividad de las personas (Por ejemplo: la casa, el trabajo, un centro de estudios, un supermercado, un centro comercial, un estadio de fútbol, una parada de bus, etc, etc, etc…). Nuestro objetivo es medir estos espacios de actividad y comprender de qué manera han variado antes y después de la pandemia COVID-19 para contribuir a mejorar la movilidad, la planificación urbana y el transporte en la ciudad de Quito.

Si luego de haber leido el párrafo anterior áun considera no haber sido informado, su participación en el proyecto no se podrá considerar. Si por el contrario, consiente haber comprendido la forma en que se usará su información, le invito a regresar a la pregunta anterior y seleccionar la opción SI para acceder a la encuesta.

Agradezco mucho su colaboración

Giovanny Moncayo

Google no creó ni aprobó este contenido.

Google Formularios
